# Case report: a 58 -year -old man with small kidneys and elevated liver enzymes

**DOI:** 10.1186/s12882-020-01762-4

**Published:** 2020-03-27

**Authors:** Jonathan Dash, Patrick Saudan, Ariane Paoloni-Giacobino, Solange Moll, Sophie de Seigneux

**Affiliations:** 1 Service of Internal Medicine, Department of medicine, University Hospital of Geneva, Geneva, Switzerland; 2 Service of Nephrology, Department of Medicine, University Hospital of Geneva, Geneva, Switzerland; 3 Service of medical Genetics, Department of medical Diagnosis, University Hospital of Geneva, Geneva, Switzerland; 4 Service of Pathology, Department of Medical Diagnosis, University Hospital of Geneva, Geneva, Switzerland

**Keywords:** Karyomegalic interstitial nephritis, Genetic, Chronic kidney disease

## Abstract

**Background:**

The conjunction of hepatitis and renal disease can be seen in several clinical context, including karyomegalic nephritis (KIN). Karyomegalic nephritis (KIN) is a rare genetic disease, with less than 50 cases reported, which incidence is probably underestimated. We report here an unusual case presentation of KIN with obtention of several organ biopsies and a novel mutation leading to the disease.

**Case presentation:**

A 58 year old Caucasian without relevant family history presents with advanced chronic kidney disease, elevated liver enzymes and recurrent pulmonary infection. Familial history was negative. Renal biopsy revealed a chronic tubulo-intertsitial nephritis with enlarged and irregular hyperchromatic nuclei. Karyomegalic nephritis (KIN) was confirmed by genetic testing with a non-sense mutation and a deletion in the Fanconi anemia associated nuclease 1 (*FAN1*) gene.

**Conclusions:**

KIN is rare disease to be suspected in the presence of renal disease, biological hepatitis and recurrent pulmonary infections, even without a familial history. Diagnosis of this condition is crucial to perform family screening, avoid progression factors, and adapt post transplantation immunosuppression. Finally, avoiding familial heterozygote donors appears of major importance in this condition.

## Specificity of the case

We present here a case of KIN nephritis with recessive inheritance and undescribed mutations. The other specificity of the case relies on the obtention of biopsies from several organs in the same patients, allowing to compare renal and extrarenal lesions.

## Background

Karyomegalic nephritis (KIN) is a rare genetic disease, described in 1974 for the first time. KIN is defined as a systemic disease, with presence of tubular Karyomegaly. Less than 50 cases are reported. We report here an unusual case presentation of KIN with obtention of several organ biopsies and a novel mutation leading to the disease.

## Case presentation

Initial presentation: A 58-year-old Caucasian man of Bulgarian origin was referred to our nephrology department because of progressive renal disease without hypertension or diabetes. The patient was previously known for one episode of hepatitis at age 38 of unclear origin, gastro-intestinal aspecific complaints, and recurrent pulmonary infections. He had no relevant familial or personal history of renal disease. The patient had no consanguinity in his family and reported having potentially being exposed to ionizing radiation in Ukraine during his childhood.

Initial laboratory: Serum creatinine level was 245 μmol/l, (eGFR 24 ml/min/1.73m^2^), the urinalysis was bland and proteinuria was of less than 1 g, with a minority of albumin. Except for a mild metabolic acidosis, no electrolyte disturbance was noted. A moderate elevation of liver enzymes was observed. Viral serology, serum ANCA, ANA immunofixation and free light chain levels were within the normal range.

### Additional examinations

**Fig. 1:**
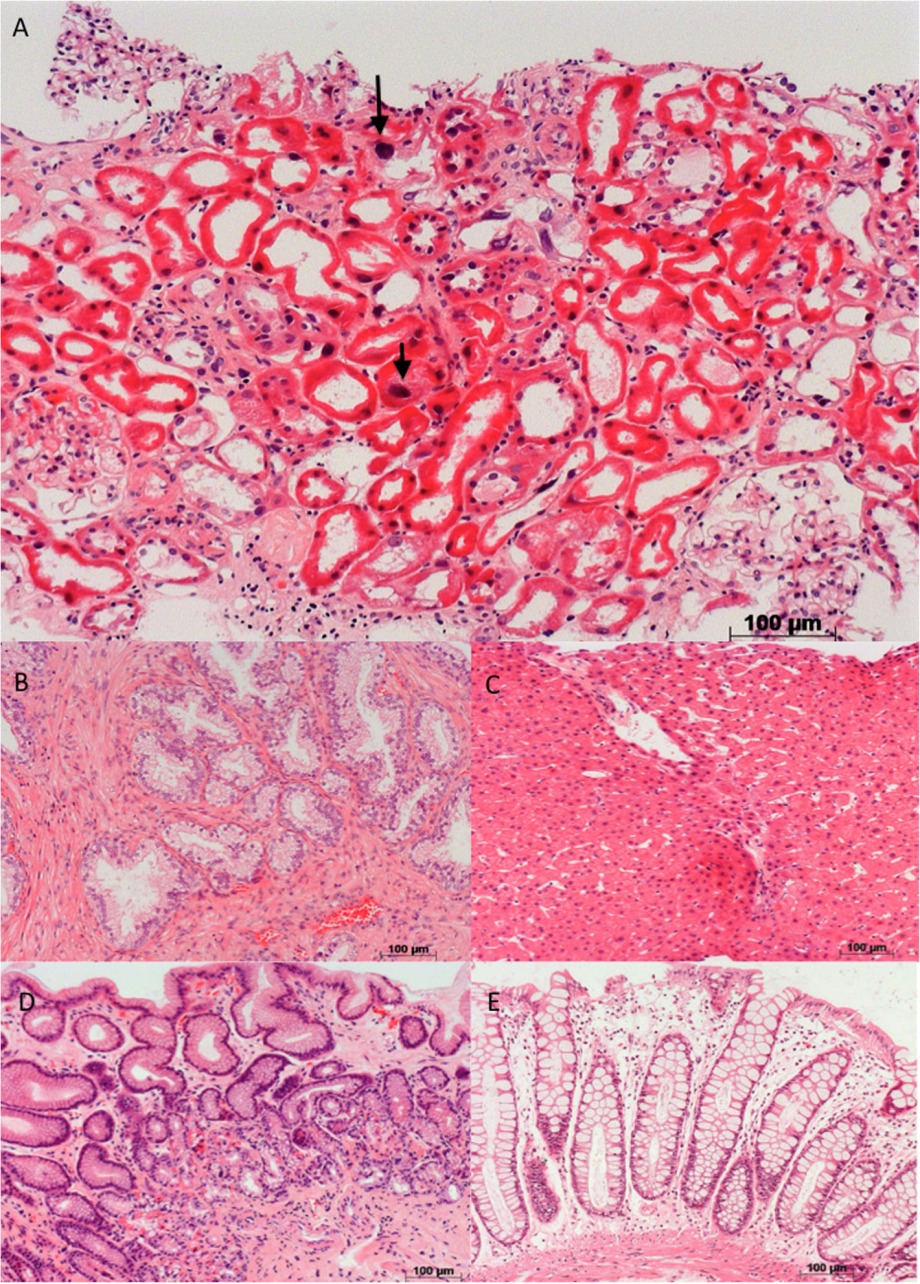
Hematoxylin and eosin staining of biopsies from the Kidney **a**, prostate **b**, liver **c**, stomach **d** and colon **e**. The arrows indicate hyperchromatic nuclei

Hepatic ultrasound was normal. A hepatic biopsy performed on account of elevated liver enzymes revealed normal hepatic parenchyma. Given intestinal complaints and a slightly elevated PSA, the patients underwent gastric, colonic and prostate biopsies which were all normal. Results for all the biopsies are displayed in Fig. 1.

A pulmonary CT scan displayed no specific anomalies, and pulmonary functions were normal.

A kidney ultrasound revealed kidneys of decreased size (Right kidney: 7.7 × 3.5 × 4.3 cm, left Kidney 7.4 × 4.5 × 4.0 cm) with a hyperechoic parenchyma and diminution of the cortico-medullary dedifferentiation. A kidney biopsy was performed and analyzed by light microscopy.

The kidney biopsy revealed 43 glomeruli, among which 23 were sclerosed. The remaining glomeruli were slightly enlarged with mesangial hypercellularity, and non-specific chronic lesions. Interstitial fibrosis including inflammatory cells (lymphocytes and histiocytes) was estimated to be around 40% of the cortical surface. The most notable anomaly was enlarged hyperchromatic nuclei in tubular cells, with irregularities, but without visualization of viral inclusions, together with the presence of tubular atrophy. These anomalies were predominant in the proximal tubules of both the cortex and the medulla. Immunofluorescence studies were negative. Additional staining for virus (CMV, EBV) were negative. Electron microscopy displayed tubular cells with signs of chronic injury, together with enlarged mitochondria in tubular cells and podocytes, but no Karyomegalic nuclei were seen on the available material.

Given the renal histological lesions in a patient with a chronic history of hepatitis and recurrent pulmonary infections, a presumptive diagnosis of Karyomegalic nephritis was retained. Genetic testing of the patient DNA by exome sequencing revealed a non-sense mutation c.1102C > T, p.(Gln368*) in exon 2 and a deletion c.2616delA, p.(Asp873Thrfs*17) in exon 12, in the Fanconi anemia associated nuclease 1 (*FAN1*) gene. Both mutations were confirmed by Sanger sequencing. The non-sense mutation had not been reported before, but was classified as pathogenic, the deletion was already reported as pathogenic, and both consequently explaining the phenotype [1]. His son presented only the c.2616delA deletion, arguing for the compound heterozygote mutations. Compound heterozygote mutations as causative for the phenotype and transmitted with an autosomal recessive mode of inheritance have already been reported [1, 2].

The diagnosis of karyomegalic nephritis related to a FAN1 mutation was retained.

Follow up: Progression towards end-stage renal disease occurred within the following year and the patients was placed on peritoneal dialysis. Peritoneal dialysis was initially difficult because of abdominal pain. A laparoscopy without biopsies was performed and revealed an inflamed peritoneum of unknown cause. The pain spontaneously resolved. The patient was also placed on a kidney transplantation waiting list.

## Discussion and conclusions

Karyomegalic nephritis (KIN) is a rare genetic disease first described in 1974 and named in 1979 by Mihatsch and colleagues describing three cases of the disease [3]. KIN is defined as a systemic disease and has a peculiar specific renal pathology displaying features common to nephronophtisis, with the presence of tubular karyomegaly [3]. Less than 50 cases have been reported and 12 families with an autosomal recessive inheritance are identified [1]. The role of *FAN1* as a causative mutated gene was reported in 2012 [1]. The FAN1 protein is required for the repair of DNA interstrand cross- links and repair of DNA damage [1, 2]. Karyomegalic nephritis is usually observed in conjunction with recurrent pulmonary infections and hepatitis, all features presented by the patient [1–3]. Kidney specificity for fibrotic lesions and more pronounced renal karyomegaly than in other organs is possibly related to the expression level of FAN1 protein, which is highest in the kidney, or to local environmental conditions [1]. Only few case reports have included biopsy of other organs [4]. In the present case, karyomegaly was kidney specific and not apparent in other organs, suggesting a predominance of renal sensitivity due to specific repair mechanism in kidney tubular cells. Participation of ioninzing radiation may have promoted the phenotype in our case.

End-stage kidney disease in KIN patients occurs usually in their 50 to 60s. Renal function in our patient declined rapidly and required dialysis. Peritoneal dialysis was implemented and the patient was inscribed on a kidney transplantation waiting list. Few reports are available on the long-term oncologic and infectious risk in these patients when placed under immunosuppression [2]. Pulmonary infections may be limiting in this context [5]. Surveillance and adaptation of immunosuppression are therefore of importance. Familial living donation in this context has to be evaluated carefully and by genetic testing. In a previous report, a recipient with KIN presented a recurrence of KIN after an ABO compatible living-related donor from his sister [6]. Heterozygous carriers might be at increased risk of CKD and a donation in heterozygous carriers should be discouraged.

Clinician have to be aware of the diagnosis of KIN and suspect it in patients with small kidneys, elevated liver enzymes and pulmonary infections. Genetic testing may give the diagnosis if performed in the first place and may avoid the need for hepatic or other biopsies. Genetic diagnosis is also of importance for familial counseling and allograft planning.

In summary, our patient presented with KIN related to a compound heterozygote mutation of *FAN1*. Mutations in this gene should be suspected in individuals with interstitial nephropathy, elevated liver enzymes and recurrent pulmonary infections. The phenotype predominates in the kidney likely because of the specificity of FAN1 protein expression, or due to possible repeated subclinical injuries to tubular cells. Heterozygous familial donors should be avoided for transplantation.

## Data Availability

Not Applicable.

## Abbreviations

ANA: Antinuclear Antibody
ANCA: Antineutrophil Cytoplasmic Antibodies
CKD: Chronic Kidney Disease
CMV: Cytomegalovirus
DNA: Deoxyribonucleic acid
EBV: Epstein-Barr Virus
eGFR: Estimated Glomerular Filtration Rate
FAN1: Fanconi anemia associated nuclease 1
KIN: Karyomegalic interstitial Nephritis
PSA: Prostate-specific antigen
